# Acute Effects of Nicotine Amplify Accumbal Neural Responses during Nicotine-Taking Behavior and Nicotine-Paired Environmental Cues

**DOI:** 10.1371/journal.pone.0024049

**Published:** 2011-09-22

**Authors:** Karine Guillem, Laura L. Peoples

**Affiliations:** 1 Université de Bordeaux, Institut des Maladies Neurodégénératives, Bordeaux, France; 2 Centre National de la Recherche Scientifique, Institut des Maladies Neurodégénératives, Bordeaux, France; 3 Department of Psychiatry, University of Pennsylvania School of Medicine, Philadelphia, Pennsylvania, United States of America; 4 Department of Pharmacology and Physiology, Drexel University College of Medicine, Philadelphia, Pennsylvania, United States of America; VU University, Netherlands

## Abstract

Nicotine self-administration (SA) is maintained by several variables, including the reinforcing properties of nicotine-paired cues and the nicotine-induced amplification of those cue properties. The nucleus accumbens (NAc) is implicated in mediating the influence of these variables, though the underlying neurophysiological mechanisms are not yet understood. In the present study, Long-Evans rats were trained to self-administer nicotine. During SA sessions each press of a lever was followed by an intravenous infusion of nicotine (30 µg/kg) paired with a combined light-tone cue. Extracellular recordings of single-neuron activity showed that 20% of neurons exhibited a phasic change in firing during the nicotine-directed operant, the light-tone cue, or both. The phasic change in firing for 98% of neurons was an increase. Sixty-two percent of NAc neurons additionally or alternatively showed a sustained decrease in average firing during the SA session relative to a presession baseline period. These session decreases in firing were significantly less prevalent in a group of neurons that were activated during either the operant or the cue than in a group of neurons that were nonresponsive during those events (referred to as task-activated and task-nonactivated neurons, respectively). Moreover, the session decrease in firing was dose-dependent for only the task-nonactivated neurons. The data of the present investigation provide supportive correlational evidence for two hypotheses: (1) excitatory neurophysiological mechanisms mediate the NAc role in cue-maintenance of nicotine SA, and (2) a differential nicotine-induced inhibition of task-activated and task-nonactivated neurons mediates the NAc role in nicotine-induced amplification of cue effects on nicotine SA.

## Introduction

Based on human and animal research, nicotine self-administration (SA) is maintained by reinforcing properties of sensorimotor and environmental cues paired with nicotine and nicotine-taking behavior. Ongoing SA is also acutely strengthened by nicotine-induced amplification of the reinforcing properties of the cues [1], [2], [3], [4], [5], [6], [7], [8], [9], [10], [11], [12], [13]. Understanding the neural mechanisms that mediate the effects of cues and nicotine on nicotine SA is relevant to identifying causes and treatment of nicotine addiction.

In rats, disruption of NAc function decreases nicotine-conditioned place preference [14], [15] and nicotine SA [16], [17]. Blockade of normal NAc function also decreases the activating and energizing effects of conditioned stimuli [18], [19], [20], cue-reinforced drug seeking [21] and the amplifying effects of addictive drugs on the energizing and reinforcing properties of conditioned cues [20], [22], [23], [24], [25]. These and other lines of evidence [26] implicate NAc involvement in both cue and nicotine maintenance of nicotine SA.

The neurophysiological mechanisms that underlie the role of the NAc in nicotine SA are not known, but clues to the nature of these mechanisms can be found in imaging and neuropsychopharmacological studies. Human imaging studies demonstrate that nicotine-associated cues increase the blood oxygenation level-dependent (BOLD) signal in the ventral striatum [27]. In the rat, nicotine increases NAc glutamate, and blockade of NAc glutamate transmission decreases nicotine SA and cue-evoked nicotine seeking [28], [29], [30], [31], [32], [33], [34], [35]. In addition, nicotine elevates the level of NAc DA [36], [37], [38], which is a neurochemical that mediates drug-induced amplification of the reinforcing properties of conditioned cues by nicotine and other drugs [22], [39], [40]. Interestingly, DA affects NAc neuron firing in an activity-dependent manner, having no effect on or amplifying the excitability of neurons activated at the time of DA exposure and suppressing the excitability of nonactivated neurons [41], [42], [43]. Given these observations, one can hypothesize that cue maintenance of nicotine SA is mediated by cue-evoked increases in NAc neuron firing. Moreover, nicotine amplification of cue effects on nicotine SA is potentially mediated by an absolute increase in the strength of the excitatory cue-induced neuronal responses (absolute amplification hypothesis), a suppression of the activity of neurons that are nonresponsive to the cues (relative amplification hypothesis), or both. The goal of the present investigation was to test for changes in NAc firing patterns consistent with these hypotheses.

Rats were trained to self-administer nicotine according to a fixed-ratio 1 (FR1) schedule of reinforcement: Rats were intravenously infused with nicotine each time the animals pressed a lever. The drug infusion was paired with a combined light-tone cue. Chronic electrophysiological procedures were used to test for a phasic increase in the firing rate of single neurons during the light-tone cue. Given evidence that nicotine SA is maintained by sensorimotor cues concurrent with drug taking, as well as by environmental cues paired with nicotine exposure, we also characterized firing patterns during the lever-press operant (nicotine-taking behavior). Additional recordings were conducted to test for (1) a nicotine-induced increase in the strength of NAc phasic responses during the drug-taking behavior and the light-tone cue and (2) a greater nicotine-induced decrease in overall, average firing of neurons nonresponsive during the operant and cue compared to neurons that were phasically activated during those events.

## Materials and Methods

### Subjects

Eighteen male Long-Evans rats were used in the present investigation. All rats were restricted to 15–20 g of rat chow each day to maintain body weight between 360 and 380 g. Rats were maintained on a reverse light cycle (lights off: 8∶30 AM; lights on: 8∶30 PM). All handling, training, and experimental sessions were conducted in the dark phase. Rats were handled each day during the week before and after surgery and on all subsequent days of the experiment. Animals were assigned to either of two treatment groups: (1) a nicotine SA group or (2) a sucrose SA group. Protocols were conducted in accordance with the Guide for the Care and Use of Laboratory Animals published by the U.S. Public Health Service and approved by the Institutional Animal Care and Use Committee (IACUC, protocol 802681), Office of Regulatory Affairs, University of Pennsylvania, Philadelphia, PA, 19104.

### Surgery and postoperative maintenance

Prior to the start of surgery, animals were deeply anesthetized with ketamine and xylazine (5 mg/kg IP). Anesthesia was maintained with isoflurane. An indwelling catheter was surgically implanted into the external jugular vein of rats assigned to the nicotine SA group. The catheter was secured to the vein with surgical silk sutures and passed subcutaneously to the top of the back where it exited into a connector (modified 22 gauge cannula). Arrays of 16 Teflon-coated stainless steel microwires were implanted in the NAc of all animals [anterior-posterior: +0.7 to +2.7 mm; medial-lateral: ±0.8 to ±2.2 mm, relative to bregma; dorsoventral: −6.8 to −7.2 mm relative to the level skull] [44] along with a stainless steel ground wire. After surgery, animals were flushed daily with 0.2 ml of an ampicillin solution (0.1 g/ml) containing heparin (300 IU/ml) to maintain patency. Animals had free access to water but were restricted to 15 to 20 g of food each day to maintain body weight at ∼370 grams. A detailed description of the surgical and postoperative procedures is provided in other reports [45], [46], [47].

### Chronic extracellular recording procedures

Voltage signals from each microwire were recorded, amplified up to 32000×, processed, and digitally captured using commercial hardware and software (Plexon, Inc, Dallas, TX). Single units were discriminated off-line with principal component analysis (Offline Sorter, Plexon, Inc, Dallas, TX). The quality of recorded units was ensured with an interspike interval criterion (at least 97% of all interspike intervals >1900 µs) and a signal∶noise criterion (valley-peak amplitude of waveform was >3× noise band). Electrophysiological data were analyzed using NeuroExplorer (Plexon) and Matlab (Mathworks, Natick, MA).

### Apparatus

Behavioral procedures were carried out in operant chambers housed inside sound–attenuating cubicles. Chambers were equipped with a retractable lever, a houselight mounted on the ceiling, a signal light above the response lever, a white noise generator, and a tone generator. Operant equipment, hardware, and control software were purchased from Med-Associates, Inc. (St. Albans, VT).

### Experimental procedures

#### Overview

After 1 week of recovery from surgery, the rats were habituated to a tethering system used to connect the subjects to the intravenous infusion pump and electrophysiological recording apparatus during nicotine SA and recording sessions during 3 habituation sessions. Subsequent to the habituation sessions, animals were trained to self-administer either nicotine (*n* = 10) or sucrose (*n* = 8) under an FR1 schedule of reinforcement in daily SA sessions for 3 weeks. Thereafter, three electrophysiological recording sessions were interspersed among continued FR1 SA training sessions. These electrophysiological sessions included an FR1 SA session, a cue-probe session, and a dose-response session (described below).

#### Habituation sessions

Training began with 3 habituation sessions. During each daily habituation sessions, the rats were placed in a nonilluminated (dark) operant chamber for 4 h and connected via a cable to a counterbalanced fluid/electronic swivel.

#### Nicotine FR1 SA sessions

**Figure 1:**
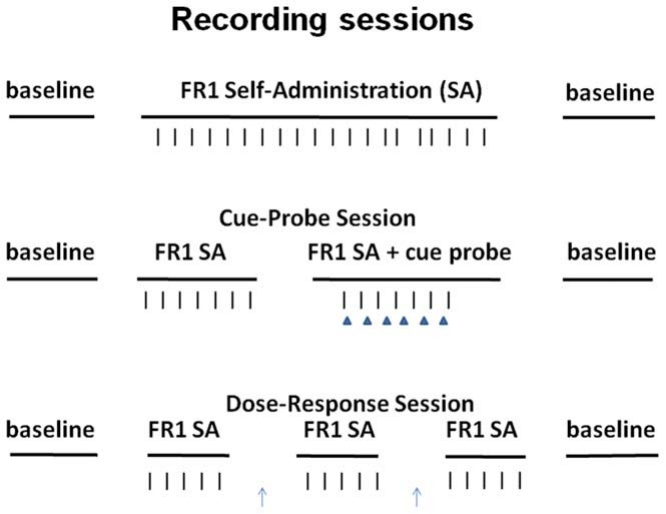
Diagram of recording sessions. Recordings were conducted during each of three types of sessions: an FR1 SA session (top), a cue-probe session (middle), and a dose-response session (bottom). The horizontal lines in each panel represent a phase of the session (not to scale). Vertical ticks correspond to reinforced lever presses (number of presses shown in figure is not representative of actual number or timing of presses). In the middle panel, the solid triangles represent presentations of the cue probe. In the bottom panel, the upward-pointing errors represent a change in the unit dose of nicotine available to the subject for self-administration. The events of each session are detailed in MATERIALS AND METHODS.

Prior to the start of each daily *nicotine FR1 SA session* (Figure 1), animals were placed in the operant chamber for a 60-min presession baseline phase. During this phase, the chamber remained dark and the response lever was retracted. The start of the SA session was signaled by illumination of a houselight and insertion of the response lever. Each time a rat pressed the lever, the subject was immediately infused with nicotine (30 µg/kg free base in 0.2 ml over 7.5 s) (press referred to as a reinforced press). The infusion was paired with a 10-s tone, a 10-s illumination of the light above the lever, and retraction of the lever. A 60-s time-out preceded reinsertion of the lever and the start of the next trial. At the end of the 2-h session, the houselight was extinguished and the lever was retracted. The animals remained in the dark chamber for 60 min.

#### Sucrose FR1 SA sessions

Sucrose FR1 SA sessions were conducted as were the nicotine SA sessions, except for the following: First, during the sucrose sessions, each press was followed by the delivery of a 32% sucrose solution into a drinking well (0.2 ml over 10 s) rather than delivery of an intravenous nicotine infusion. Second, for each subject in the sucrose group, the number of sucrose reinforcers was matched daily to the number of nicotine reinforcers earned by a paired animal in the nicotine group.

#### Cue-probe session

*A* cue-probe session was conducted to control for the possibility that the changes in firing during the light-tone cue were potentially related to the offset of the operant rather than to the cue [48], [49]. During the cue-probe session (Figure 1), rats completed 40 reinforced presses. Each press was paired with the normal light-tone cue. An additional 15 presentations of the 10-s light-tone cue were interspersed among the last 30 reinforced presses: One additional cue presentation occurred 2 min after every other press. All other aspects of the session were the same as for a normal FR1 SA training session.

#### Dose-response session

A dose-response session was conducted as part of the characterization of the acute pharmacological effects of self-administered nicotine (Figure 1). During the session, animals were exposed to 3 doses of nicotine (20, 40, and 60 µg/kg/inf) that spanned the range of doses reliably self-administered by rats [45], [50], [51], [52]. Doses were administered in either ascending or descending order, counterbalanced across animals. Each dose was maintained until the animals earned 15 infusions. Neural data collected during the period that lapsed between the 6^th^ and 15^th^ infusions (last 10 infusions at each dose) were included in data analyses.

### Categories of NAc firing patterns

#### Phasic changes in firing rate during the nicotine-taking behavior and the nicotine-paired light-tone cue

**Figure 2:**
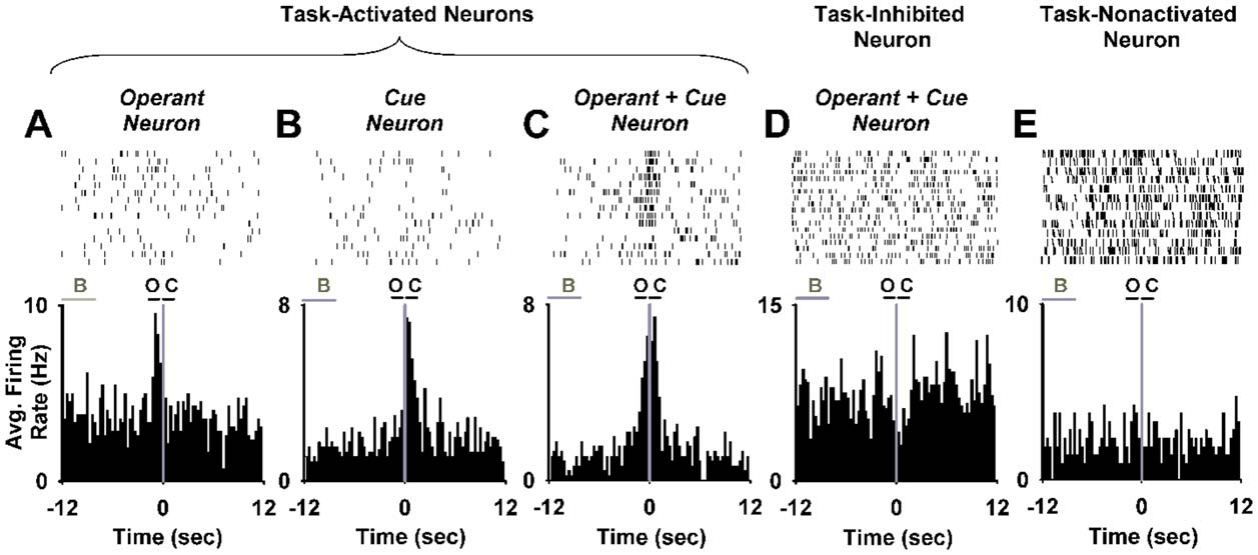
Individual neuron examples of phasic firing patterns time-locked to the nicotine-reinforced press. (**A**) Increase in firing rate during the operant but not during the cue (operant neuron). (**B**) Increase in firing during the light-tone cue but not during the operant (cue neuron). (**C**) Increase in firing during both the operant and the light-tone cue (operant + cue neuron). (**A–C**) Task-activated neurons. (**D**) Decrease in firing during the operant and the light-tone cue (task-inhibited, operant + cue neuron). (**E**) Task-nonactivated neuron. (**A–E**) At the bottom of each panel, a histogram shows the average firing rate (average Hz per 300-ms bin) of a single neuron plotted during the 12 s before and after the completed nicotine-reinforced press. Time zero on the abscissa corresponds to offset of the operant and the onset of the light-tone cue. The three horizontal lines shown at the top of the histogram at −12 to −9 s prepress, -1 to 0 s prepress, and 0 to +1 s postpress demarcate the prepress period (B), the operant period (O), and the cue period (C), respectively. Above each histogram is shown the single-trial raster for the same neuron represented in the histogram.

To characterize NAc neural responses during drug-taking behavior (lever-press operant) and the light-tone cue, individual neurons were tested for a change in firing that was time-locked to offset of the reinforced press and the concurrent onset of the light-tone cue (Figure 2). To test for the changes in firing, the average firing rates during the 1 s before (operant period) and after (cue period) completion of the press were compared separately to the average firing rate during a prepress period (−12 to −9 s before the press) using a Wilcoxon test. Based on findings of prior cocaine SA experiments [53], [54], [55], neurons were additionally tested for changes in firing during the minutes before and after reinforced presses. These tests were negative and are therefore described only in supporting information (Figure S1).

#### Session changes in average firing rate

**Figure 3:**
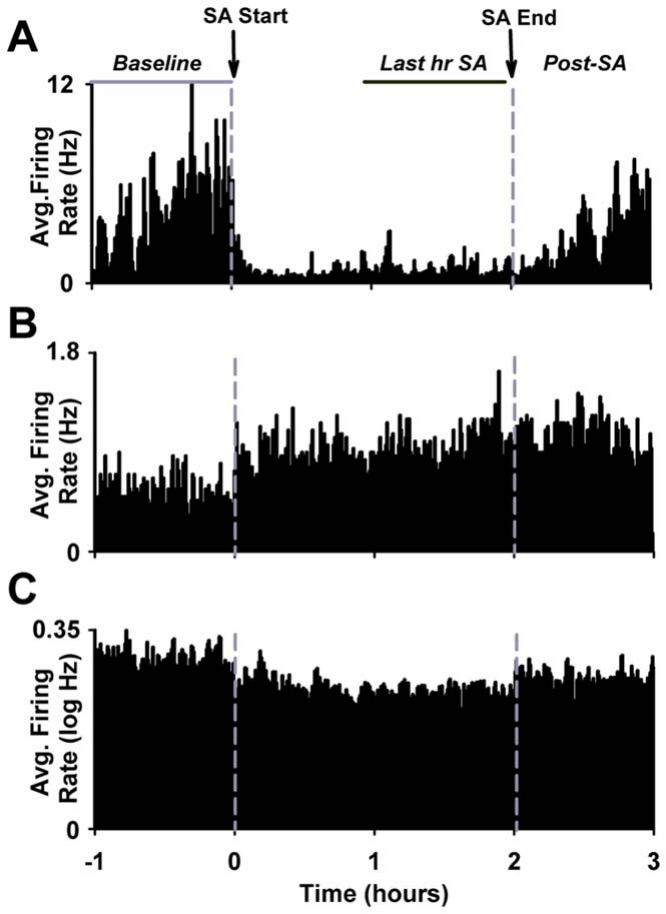
Session changes in average firing during the FR1 nicotine SA session. (**A–B**) Single-neuron example of a session decrease in firing (A) and a session increase in firing (B). In panels A and B, firing rate (Hz per 30-s bin) of a single neuron is plotted as a function of time (h) during the recording session. (**C**) Average firing rate exhibited by the entire population of recorded NAc neurons during the FR1 nicotine SA session. In panel C, the average firing rate (average log Hz per 30-s bin) of all recorded neurons is plotted as a function of time (h). In all histograms, dashed vertical lines correspond to the start and end of the SA phase of the recording session.

Individual neurons were tested for a significant and stable increase or decrease in average firing during SA relative to the drug-free, presession, baseline phase (session change in firing, Figure 3) [56], [57]. Firing rate (Hz) was calculated as a function of 30-s bins across both the 60-min presession baseline phase and the last hour of the SA session. A between-phase comparison of average firing was made using a Mann-Whitney test. If a significant difference in firing was observed, an additional analysis was conducted to characterize the stability of the change in firing. For the last hour of the SA session, the direction of the difference in average firing between each 30-s bin and the average presession firing rate was determined. If the direction of the difference was consistent with the outcome of the significance test for more than 90% of the bins, the change was defined as stable and the neuron was said to exhibit a session change in firing.

### Prevalence and magnitude of NAc firing patterns

For each animal and recording session, prevalence and magnitude measures were calculated for the phasic responses time-locked to the reinforced press. To characterize prevalence, we determined the percent of neurons showing the firing patterns on a per rat basis (percent of recorded neurons per animal). The magnitude of each firing pattern was calculated using the ratio of *S−B/S+B*, where *S* equaled firing rate during a ‘signal’ period and *B* equaled firing rate during the prepress period −12 to −9 s prepress). The signal period was defined as (1) the operant period for neurons responsive exclusively during the operant (Figure 2A); (2) the cue period for neurons responsive exclusively during the cue (Figure 2B); and (3) the combined operant and cue periods for neurons responsive during both the operant and the cue (Figure 2C). Comparable procedures were used to characterize the prevalence and magnitude of session changes in firing.

### Task-activated versus task-nonactivated neurons

To test the relative amplification hypothesis, we compared the effect of nicotine on two subtypes of neurons: those activated during the operant and light-tone cue (task-activated neurons, Figure 2A–C) and those nonresponsive during the same events (task-nonactivated neurons, Figure 2E). All session-increase neurons were excluded from this comparison, given evidence in the present investigation that session increases in average firing are nonpharmacological.

It was possible that the response of task-activated neurons to the acute pharmacological effects of nicotine varied depending on whether the phasic increase was related to either the light-tone cue or the drug-taking behavior. This possibility was investigated in the cue-probe session, during which task-activated neurons were sorted into two groups: (1) those responsive during the operant but not during the cue probe (probe nonresponsive) and (2) those responsive during the light-tone cue post-press and the cue-probe (probe responsive). The average firing rates of the task-activated subgroups and the task-nonactivated neurons were compared during the presession baseline and SA phases of the cue-probe session.

### Statistical analyses of neural firing

Group mean comparisons were conducted with analyses of variance (ANOVA). To reduce the skewness of the distribution of individual neuron firing rate data for ANOVA analyses, data were transformed (log10 [x + 1], where x = Hz) (Peoples et al., 2004, 2007). All average values were reported as mean ± the standard error of the mean (± SEM). Preliminary ANOVA analyses showed no significant differences between neurons activated during the operant versus neurons activated during the light-tone cue. The two groups were combined in most analyses presented in **Results** to simplify presentation.

### Histological Analysis

**Figure 4:**
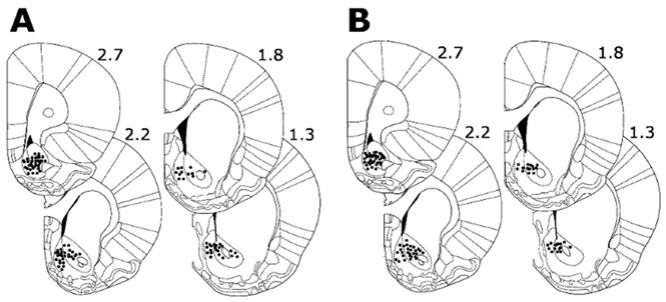
Locations of individual wire tips in the NAc. (**A–B**) The locations at which NAc neurons were recorded during the FR1 nicotine SA session (A) and the FR1 sucrose SA session (B). Numbers indicate millimeters anterior to bregma.

Histological procedures were used to identify the location of all wire tips used to record neurons. With the animals under anesthesia, anodal current (50 µA for 5 s) was passed through each microwire. Animals were then perfused with 4% paraformaldehyde in 0.9% saline. The brains were cut into 50-µm coronal sections that were mounted on slides and incubated in a solution of 5% potassium ferricyanide and 10% hydrochloric acid to stain the iron deposits left by the recording tips. The tissue was counterstained with a 0.2% solution of neutral red. The location of each wire tip was plotted on the coronal plate [44] that most closely corresponded to its anterior-posterior position. Neurons recorded from wires that were not within the boundaries of the NAc were excluded from all analyses. Preliminary ANOVA analyses showed that the prevalence of core and shell neurons [58] was comparable between the sucrose and nicotine groups (Figure S2A). Moreover, within each of the sucrose and nicotine groups, there was no effect of subterritory (core *vs* shell) on the prevalence of task-activated versus task-nonactivated neuron groups (Figure S2B). Finally, for the nicotine group, the prevalence of core neurons and shell neurons was stable across the three recording sessions (Figure S2C). The locations of NAc neurons recorded during the FR1 SA session are shown in Figure 4.

## Results

### Nicotine group

#### Nicotine SA behavior

Prior to the first recording session, the average interpress interval (intertrial interval, ITI) during the nicotine SA session varied less than 10% for 3 consecutive days. During the FR1 nicotine SA recording session, rats earned an average of 33±1.59 nicotine infusions during the 2-h SA phase; average ITI for the last 15 self-infusions equaled 7.63±0.75 min. A similar rate of drug intake occurred during the cue-probe session (30.2±0.73 infusions in 2 h; average ITI for last 15 self-infusions = 7.77±0.95 min). Consistent with the findings of previous investigators [52], increasing the nicotine dose during the dose-response session moderately decreased the average rate of nicotine intake (average ITI for the last 10 self-infusions = 6.65±1.10 min, 6.87±1.18 min, and 9.36±1.69 min for the 20, 40, and 60 µg/kg/inf doses; *F*_(2,18)_ = 0.85; NS).

#### Phasic changes in firing during the nicotine-taking behavior and the nicotine-paired light-tone cue

During the *FR1 nicotine SA session* (Figure 1), 19.5% (17/87) of neurons exhibited an increase in firing time-locked to the reinforced press (phasic-increase neurons) (Figure 2A–C). Two percent (2/87) showed a decrease in firing (phasic-decrease neurons) (Figure 2D). Of the phasic-increase neurons, 30% (5/17) responded exclusively during the press (operant neurons); 47% (8/17) responded exclusively during the light-tone cue (cue neurons); and 23% (4/17) responded during both the operant and the cue (operant + cue neurons) (Figure 2A–C).

**Figure 5:**
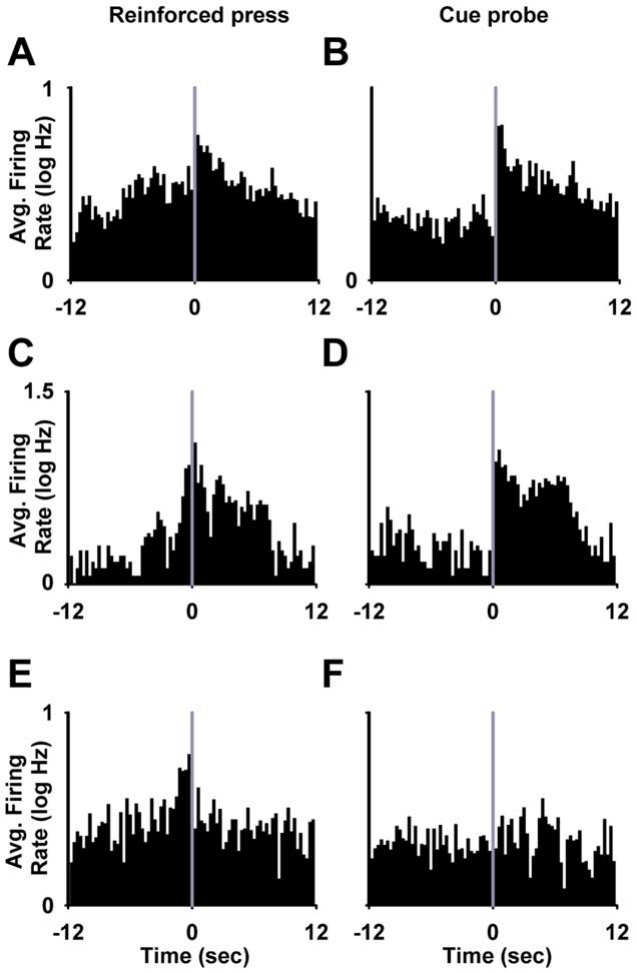
Phasic firing of NAc neurons during the cue-probe session. (**A–F**) Group mean histograms showing the average firing of cue neurons (A, B), operant + cue neurons (C, D), and operant neurons (E, F) time-locked to the nicotine-reinforced press (panels A, C, and E) and to the cue probe (panels B, D, and F). In each histogram, the average firing rate (average log Hz per 300-ms bin) is plotted for the 12 s before and after either the nicotine-reinforced press (A,C, and E) or the cue probe (B, D, and F).

During the *cue-probe session* (Figure 1), 22% (20/89) of neurons exhibited a phasic increase in firing. Of those 20 neurons, 20% (4/20) were operant neurons; 55% (11/20) were cue neurons; and 25% (5/20) were operant + cue neurons. None of the operant neurons showed a response during the cue probe. However, a majority of the cue neurons (7/11) and all operant + cue neurons (5/5) increased firing during the cue probe (Figure 5). The average firing rate of the cue and operant + cue neuron groups increased significantly between the −12 to −9 s preprobe and the first 1 s of the cue probe (0.34±0.04 log Hz to 0.72±0.10 log Hz, *F*_(1,9)_ = 11.28; *p*<0.01; and 0.33±0.4 log Hz to 0.77±0.17 log Hz, *F*_(1,4)_ = 10.91; *p*<0.05).

During the cue-probe session, three neurons showed a phasic decrease in firing time-locked to the reinforced press (1 cue neuron and 2 operant + cue neurons). None of the three neurons showed a change in firing during the cue probe. In addition, neurons that showed no response time-locked to the reinforced operant also showed no change in firing during the cue probe.

**Figure 6:**
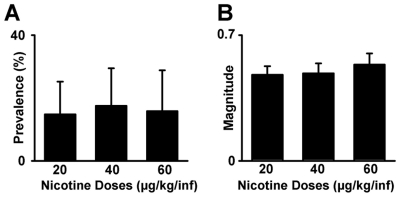
Phasic firing during the dose-response session. (**A**) Average prevalence of neurons (average number of neurons per subject) that showed a phasic increase in firing time-locked to the nicotine-reinforced press is plotted for each nicotine dose. (**B**) Average magnitude of all phasic increases in firing time-locked to the nicotine-reinforced press is plotted as a function of nicotine dose. The magnitude of each firing pattern was calculated using the ratio of *S−B/S+B*, where *S* equaled firing rate during a ‘signal’ period and *B* equaled firing rate during the prepress period (−12 to −9 s prepress). The signal period was defined as the operant period for neurons responsive exclusively during the operant (Figure 2A), the cue period for neurons responsive exclusively during the light-tone cue (Figure 2B), and the combined operant and cue periods for neurons responsive during both the operant and the light-tone cue (Figure 2C).

During the *dose-response session* (Figure 1), the prevalence of neural responses to the operant and cue (Figure 6) were similar to those during the FR1 SA and cue-probe sessions. In addition, the prevalence and the magnitude of the phasic increases were not affected by nicotine dose. A repeated-measures ANOVA with dose as a factor (20, 40, and 60 µg/kg/inf) showed no significant effect of dose on either the prevalence (*F*_(2,16)_ = 0.07; NS) (Figure 6A) or the average magnitude (*S−B/S+B*, see **Materials and Methods** **: Prevalence and magnitude of NAc firing patterns**) (*F*_(2,14)_ = 0.35; NS) (Figure 6B) of phasic-increase firing patterns. Comparable findings were obtained when analyses were conducted with all phasic-increase neurons sorted into two groups: those that showed a response during the operant period and those that showed a response during the cue period (Figure S3). The number of neurons showing a phasic decrease in firing time-locked to the press remained low across all nicotine doses (0–3 neurons at each dose).

**Figure 7:**
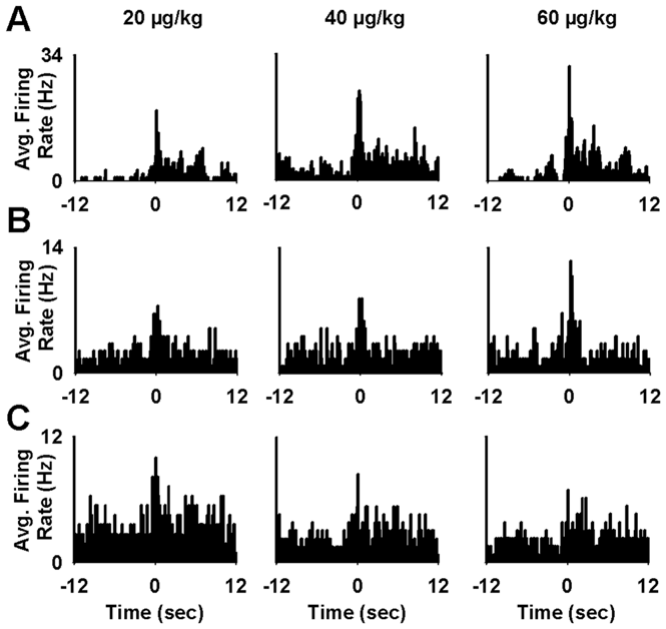
Individual neuron examples of changes in phasic responses that occurred in association with increases in nicotine dose. (**A**) Increase in response magnitude associated with an increase in signal. (**B**) Increase in response magnitude associated with a combined increase in signal and decrease in background. (**C**) Decrease in response magnitude associated with a decrease in signal. (**A–C**) Each row shows the firing patterns of a particular single neuron at each of the three nicotine doses (20, 40, and 60 µg/kg/inf = left, middle, and right column, respectively). In each histogram, average firing rate of an individual neuron (average Hz during the last 10 trials at each dose) is plotted for the 12 s pre- and postpress.

A descriptive individual neuron analysis of phasic increases during the *dose-response session* showed diverse responses to nicotine dose (Figure 7A–C). Between 20 and 60 µg/kg nicotine, 37% of phasic-increase neurons exhibited less than a 20% change in response magnitude (average change = 7%±4%); 13% showed a greater than 20% decrease (average change = 31%±2%) (Figure 7C); and 50% showed a greater than 20% increase (average change = 66%±9%). For the latter group of neurons, the change in response magnitude reflected either an increase in firing during the signal period (40% of neurons, Figure 7A) or both an increase in firing during the signal period and a decrease in firing during the −12 to −9 s prepress period (60% of neurons, Figure 7B). The majority of neurons showing a ≥20% change in response magnitude were cue neurons (75% cue, 25% operant, and 0% operant + cue).

#### Session changes in average firing

During the *FR1 SA recording session*, 62% (54/87) of neurons exhibited a sustained decrease in firing rate (Hz per 30-s bin) during the SA phase relative to the presession baseline phase (session-decrease firing pattern) (Figure 3A). Fifteen percent of neurons (13/87) showed a sustained increase (session-increase firing pattern) (Figure 3B). Between-group ANOVAs with firing pattern (session-increase *vs* session-decrease) as a factor showed that session decreases were significantly more prevalent than session increases (*F*_(1,18)_ = 46.76; *p*<0.001); however, the average magnitude of the session changes in firing did not differ significantly (*F*_(1,72)_ = 1.98; NS). Consistent with the greater prevalence of session decreases, a repeated-measures ANOVA with session phase as a factor showed that the average firing rate of all recorded neurons decreased significantly between the presession baseline phase and the nicotine SA phase (0.30±0.03 *vs* 0.24±0.02 log Hz; *F*_(1,86)_ = 11.58 ; *p*<0.01) (Figure 3C).

**Figure 8:**
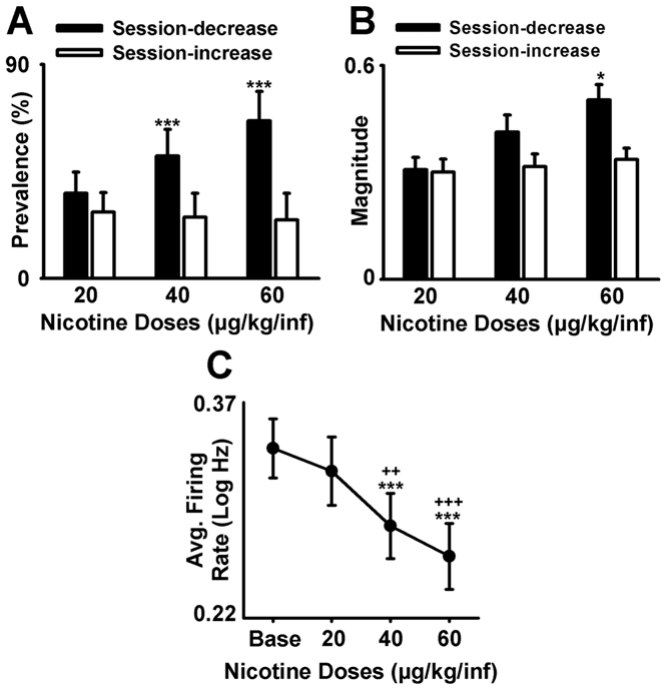
Average firing-rate changes during the nicotine dose-response session. (**A**) Average prevalence of neurons (average number of neurons per subject) that showed either a decrease or an increase in average firing during nicotine SA is plotted as a function of nicotine dose. (**B**) Average magnitude (*S−B/S+B*) of the session-decreases and session-increases in firing rate during nicotine SA is plotted as a function of nicotine dose (signal period = last h of the SA session and baseline period = the 1-h presession baseline phase). *p<0.05 and ***p<0.001, significant difference when compared to the lower dose of nicotine (20 µg/kg/inf). (**C**) Average firing rate of the entire population of recorded NAc neurons is plotted for the presession baseline phase (Base) and each test dose of nicotine. **p<0.01 and ***p<0.001, significant difference when compared to presession baseline.

During the *dose-response session*, the prevalence and magnitude of session changes in firing varied with nicotine dose (Figure 8). A mixed-design ANOVA analysis with dose (20, 40, and 60 µg/kg/inf) and firing pattern (decrease *vs* increase) as factors showed a significant effect of dose on the prevalence (*F*_(2,32)_ = 12.77; *p*<0.001) and magnitude (*F*_(2,66)_ = 2.93; *p*<0.05) of session changes. There was also a significant firing-pattern × dose interaction effect on the prevalence (*F*_(2,32)_ = 19.51; *p*<0.001) and the magnitude (*F*_(2,82)_ = 2.63; *p*<0.05) of the session-changes in firing. Post hoc analyses showed that incrementing the nicotine dose had no significant effect on session increases in firing but significantly increased the prevalence (20 *vs* 40 and 20 *vs* 60 µg/kg/inf; *p*<0.001) (Figure 8A) and the magnitude (20 *vs* 60 µg/kg/inf; *p*<0.05) of session decreases (Figure 8B). Consistent with these findings, a repeated-measures ANOVA with dose as a factor showed a significant effect of dose on the average firing rate of all recorded neurons (*F*_(3,183)_ = 11.96; *p*<0.001) (Figure 8C). Post hoc analysis showed that increasing the nicotine dose significantly decreased the average firing of all recorded neurons (20<40 and 60 µg/kg/inf nicotine; *p*<0.01 and *p*<0.001, respectively).

#### Does the inhibitory effect of nicotine have a greater effect on task-nonactivated neurons than on task-activated neurons?

**Figure 9:**
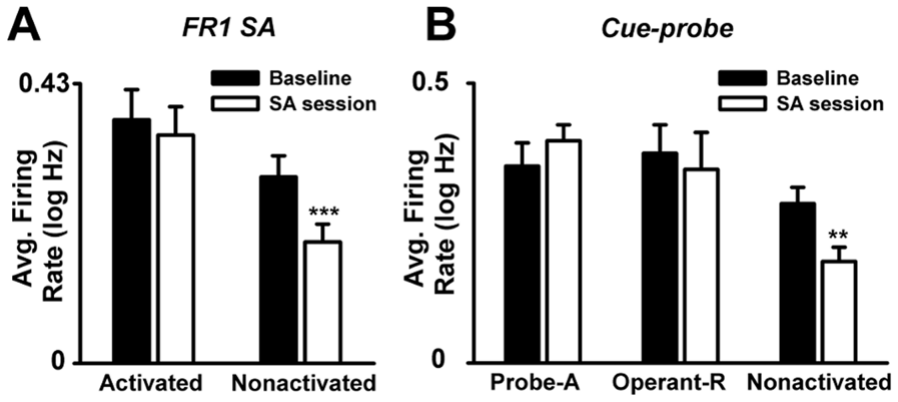
Average firing of task-activated versus task-nonactivated neurons during the FR1 nicotine SA and cue-probe sessions. (**A**) Average firing of task-activated and task-nonactivated neurons (Activated and NonActivated) during the presession baseline and SA phase of the FR1 nicotine SA session. *p<0.05, significant difference when compared to baseline. (**B**) Average firing of probe-responsive neurons (Probe-A), operant but not cue-probe-responsive neurons (Operant-R), and task-nonactivated (Non-Activated) neurons during the presession baseline and SA phases of the cue-probe session. **p<0.01, significant difference when compared to baseline.

Additional ANOVA analyses of the *FR1 SA session* tested for a greater decrease in the average firing of operant- and cue-nonresponsive neurons (task-nonactivated neurons) (Figure 2E) during nicotine SA compared to neurons that were activated during the operant and the light-tone cue (task-activated neurons) (Figure 2A–C) (see **Materials and Methods**: **Task-activated versus task-nonactivated neurons**, for additional description of analysis). These analyses showed that the average number of neurons exhibiting a session decrease was significantly greater for the task-nonactivated neuron group (82%) than for the task-activated neuron group (41%) (*z* = 3.23; *p*<0.01). There was also a trend for the average magnitude of the session-decrease firing patterns to be greater for the task-nonactivated group (0.40±0.04 log Hz) than for the task-activated group (0.21±0.04 log Hz) (*F*_(1,46)_ = 3.54; *p* = 0.07). In line with these observations, a repeated-measures ANOVA with activation (task-activated *vs* task-nonactivated) and session phase (presession baseline *vs* SA) as factors showed a significant effect of session phase (*F*_(1,66)_ = 7.54; *p*<0.01) and a significant interaction between session phase and activation (*F*_(1,66)_ = 7.24; *p*<0.01). Post hoc tests showed that average firing decreased significantly during the SA phase for the task-nonactivated neuron group (*p*<0.001) but not for the task-activated neuron group (*p*>0.05) (Figure 9A). An additional control analysis showed that the effect of activation on average firing was apparent during different behavioral periods (prepress, operant, and cue periods) and hence not attributable to a specific behavior (Figure S4A).

During the *cue-probe session*, comparisons of average firing were made among the task-nonactivated neuron group and two subgroups of task-activated neurons: probe-responsive and probe nonresponsive. A mixed-design ANOVA with activation and session phase as factors showed no significant effect of activation (*F*_(2,67)_ = 2.18; NS) and no significant effect of session phase (*F*_(1,67)_ = 0.23; NS). However, a significant interaction was noted between activation and session phase (*F*_(2,67)_ = 7.66; *p*<0.01). Post hoc analysis of this interaction showed that average firing rate during the SA phase decreased significantly relative to the presession baseline for the task-nonactivated group (*p*<0.01) but not for either of the two task-activated neuron subgroups (Figure 9B). These findings showed that probe-responsive and probe-nonresponsive task-activated neurons exhibited comparable firing rates; both subtypes of task-activated neurons also showed a smaller decrease in average firing during the SA session relative to the task-nonactivated neuron group.

**Figure 10:**
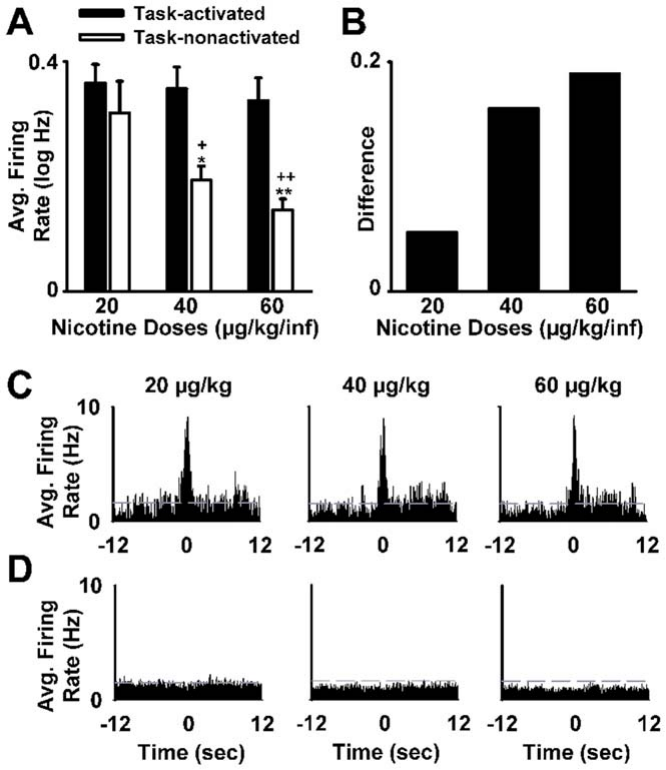
Differential decrease in average firing of task-activated versus task-nonactivated neurons during the nicotine dose-response session. (**A**) Average firing rate (log Hz) of task-activated and task-nonactivated neurons is plotted as a function of nicotine dose. *p<0.05, **p<0.01, significant difference relative to the 20 µg/kg/inf dose for the task-nonactivated neuron group. +*p*<0.05, ++*p*<0.01, significant difference between the task-activated and task-nonactivated neuron groups. (**B**) The difference in average firing rate between the task-activated and task-nonactivated group is plotted as a function of nicotine dose. (**C–D**) Each histogram shows average firing (Hz per 300-ms bin) of the task-activated neuron group (C) and of the task-nonactivated neuron group (D) during SA of one dose of nicotine (20, 40, and 60 µg/kg/inf). In each histogram, average firing rate is plotted for the 12 s before and after the nicotine-reinforced press.

During the *dose-response session*, comparisons of the task-activated and task-nonactivated neurons showed that the two groups responded differently to increases in nicotine dose. A mixed-design ANOVA of average firing with activation and dose as factors showed a significant effect of activation (*F*_(1,34)_ = 7.66; *p*<0.01), a significant effect of dose (*F*_(2,68)_ = 3.42; *p*<0.05), and a significant interaction between activation and dose (*F*_(2,68)_ = 2.65; *p*<0.05). Post hoc analyses showed that the average firing rate of the task-nonactivated neuron group decreased dose-dependently (20 *vs* 40, *p*<0.05; 20 *vs* 60, *p*<0.01), whereas the task-activated group showed no significant change in average firing as the nicotine dose was increased (Figure 10A). The dose-dependent differential decrease in average firing was associated with a dose-dependent increase in the positive difference in firing between the task-activated neuron group and the task-nonactivated group (20 µg/kg/inf, NS; 40 µg/kg/inf, *p*<0.05; 60 µg/kg/inf, *p*<0.01) (Figure 10B–D). Control analyses showed that the stability in average firing of the task-activated neuron group and the dose-dependent decrease in average firing of the task-nonactivated neuron group were apparent during different behavioral periods and thus not attributable to nicotine-induced changes in behavior (Figure S4B).

### Sucrose control group

The nicotine dose-response data support the interpretation that the pharmacological effect of nicotine on NAc neurons is an activity-dependent decrease in average firing. To conduct a further test of this interpretation, we tested for a similar decrease in average firing of NAc neurons during sucrose SA.

#### Phasic changes in firing during the sucrose-taking behavior and the sucrose-paired light-tone cue

**Figure 11:**
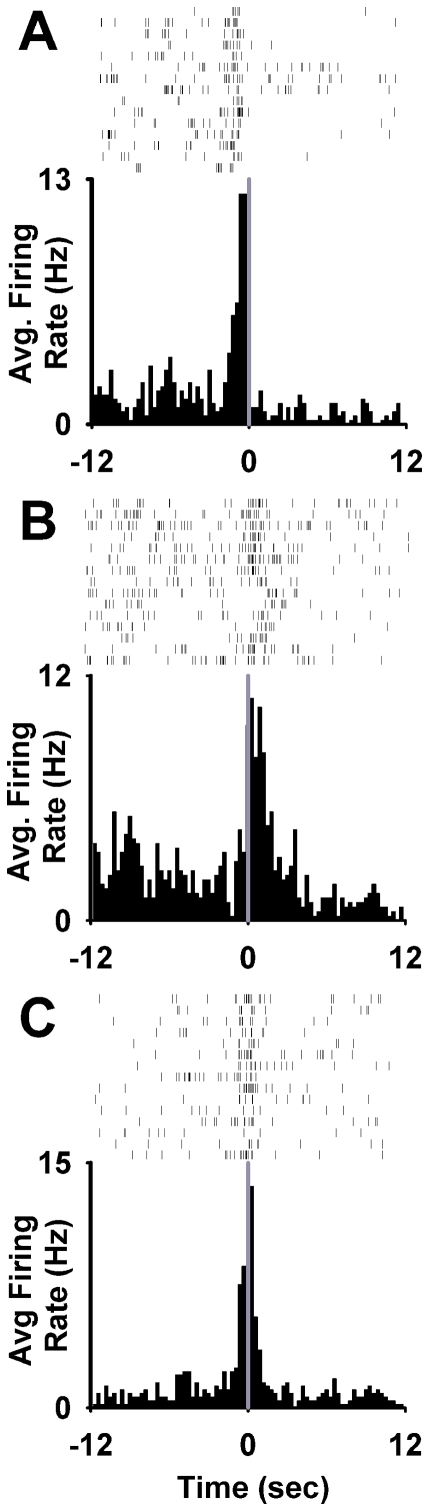
Individual neuron examples of phasic increases in firing time-locked to the sucrose-reinforced press. (**A**) Increase in firing during the operant but not during the light-tone cue (operant neuron). (**B**) Increase in firing during the light-tone cue but not during the operant (cue neuron). (**C**) Increase in firing during both the operant and the light-tone cue (operant + cue neuron). (**A–C**) At the bottom of each panel, a histogram shows the average firing rate (average Hz per 300-ms bin) of a single neuron plotted during the 12 s before and after the completed sucrose-reinforced press. Above each histogram is shown the individual trial raster for the same neuron represented in the histogram.

During the FR1 sucrose SA session, 20.3% (13/64) of the neurons exhibited a phasic increase in firing time-locked to the reinforced press. Thirty-eight percent of the phasic increases occurred exclusively during the operant period; 46% occurred exclusively during the cue period; and 16% occurred during both the operant and the cue periods (Figure 11A–C).

**Figure 12:**
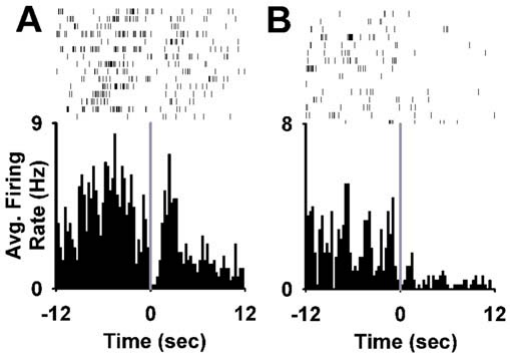
Individual neuron examples of phasic decreases in firing time-locked to the sucrose-reinforced press. (**A**) Decrease in firing during the operant and the light-tone cue (operant + cue neuron). (**B**) Decrease in firing during the light-tone cue (cue neuron). (**A–B**) At the bottom of each panel, a histogram shows the average firing rate (average Hz per 300-ms bin) of a single neuron plotted during the 12 s before and after the completed sucrose-reinforced press. Above each histogram is shown the individual trial raster for the same neuron represented in the histogram. The bottom row of the raster corresponds to the last trial of the session. Time 0 = the completed sucrose-reinforced lever press.

Twenty-three percent (15/64) of neurons exhibited a decrease in firing time-locked to the sucrose-reinforced press (Figure 12). Forty percent (6/15) of the phasic decreases occurred exclusively during the operant; 33% (5/15) occurred exclusively during the cue; and 27% (4/15) occurred during both the operant and the cue. For 6 of the neurons (6/64 = 9%), the time course of the decrease was similar to that of phasic increases (Figure 12A) and tightly time-locked to the 1 s pre- and postpress. For 9 other neurons (9/64 = 14%), the inhibitory response was sustained through the period of sucrose consumption (+2 to +12 s postpress) (Figure 12B).

#### Session changes in firing during the FR1 SA session

**Figure 13:**
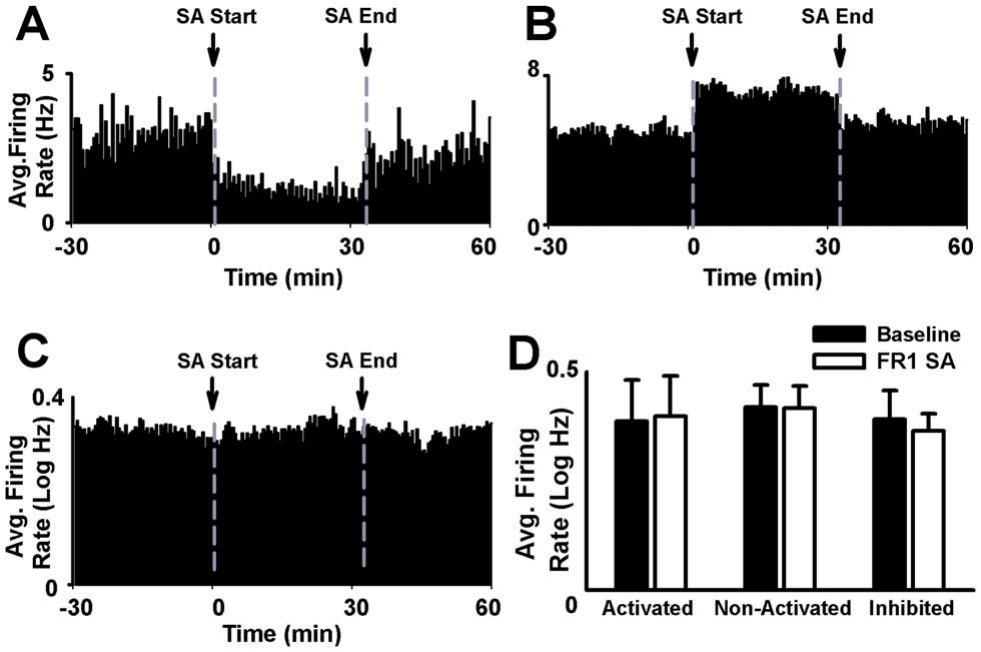
Session changes in average firing during the FR1 sucrose SA session. (**A–B**) Single-neuron example of a session decrease in firing (A) and a session-increase in firing. In both A and B panels, firing rate (Hz per 30-s bin) is plotted as a function of time (h) during the recording session. (**C**) Average firing rate exhibited by the entire population of recorded NAc neurons during the FR1 sucrose SA session. Average firing rate (average log Hz per 30-s bin) of all recorded neurons is plotted as a function of time (h) during the recording session. In all histograms, dashed vertical lines correspond to the start and end of the SA phase of the recording session. (**D**) Average firing rate (average log Hz) of task-activated (activated), task-nonactivated (nonactivated), and task-inhibited (inhibited) neuron groups during the presession baseline and the SA phase of the sucrose SA recording session.

During the FR1 sucrose SA session, 26% (16/64) of neurons showed a session decrease in firing and 20% (13/64) of neurons showed a session increase in firing (Figure 13A–B). Separate between-group ANOVAs showed no significant effect of firing pattern on either prevalence (*F*_(1,14)_ = 1.03; NS) or magnitude of the session changes in firing (*F*_(1,30)_ = 0.90; NS). Moreover, no significant change was observed in average firing during the SA session (0.37±0.04 log Hz) relative to the presession baseline (0.39±0.03 log Hz) (*F*_(1,63)_ = 0.72; NS) (Figure 13C).

Phasic-decrease firing patterns time-locked to the reinforced press were more prevalent during the sucrose FR1 SA session than during the nicotine FR1 SA session (23% *vs* 2%). Session-decrease firing patterns showed the reverse pattern of prevalence (26% *vs* 62%). Moreover, in the sucrose group, only 1/13 phasic-decrease neurons showed a session-decrease firing pattern. Given these observations, it was possible that the difference in prevalence of session decreases between the nicotine and sucrose groups was linked to the differential presence of the phasic-decrease neurons. However, exclusion of all phasic-decrease neurons from the analyses of session-change firing patterns and average firing rates in the sucrose group did not alter the outcome of those analyses (Supplementary Information **S1**: **Sucrose FR1 SA session**).

#### Task-activated versus task-nonactivated neurons

The test for an activity-dependent decrease in average firing during the sucrose FR1 SA session was comparable to that applied to the nicotine group except that phasic-decrease neurons (task-inhibited neurons) were treated as a separate activation group. A mixed-design ANOVA analysis of average firing rates showed no significant effect of activation (task-activated, task-nonactivated, and task-inhibited) (*F*_(2,61)_ = 0.50; NS), no significant effect of session phase (*F*_(1,61)_ = 0.91; NS), and no significant interaction between activation and session phase (*F*_(2,61)_ = 1.22; NS). Thus the task-activated, task-inhibited, and task-nonactivated neuron groups showed comparable average firing rates and no significant change in average firing rate during the sucrose SA session relative to the presession baseline phase (Figure 13D).

### Anatomic analysis: FR1 SA session

Analyses of the FR1 session showed that phasic increases in firing time-locked to the nicotine-reinforced press were significantly greater in magnitude in the core than in the shell, though there was no significant effect of subterritory (core *vs* shell) on prevalence of phasic firing patterns (Figure S5). The prevalence of session increases and decreases was comparable between the core and shell (Figure S6A). Moreover, the average firing rate of both shell and core neurons showed a decrease during SA relative to the presession baseline phase (Figure S6B). The magnitude of the decrease was similar between the two subterritories, though overall firing rates during the presession and SA phase were lower for the shell neurons than for the core neurons (Figure S6B). There was no significant effect of subterritory on the differential decrease in average firing exhibited by task-activated versus task-nonactivated neurons (Figure S6C). Analysis performed during the FR1 sucrose SA session showed no significant effect of subterritory on phasic firing patterns, session-change firing patterns, or the average firing of the task-activated and task-nonactivated neuron groups (not shown).

## Discussion

### Major findings

During FR1 nicotine SA, subgroups of NAc neurons increased firing during nicotine-taking behavior (lever-press operant), an environmental nicotine-paired cue (light-tone cue), or both. Over 75% of recorded NAc neurons additionally or alternatively showed a sustained change in average firing during the SA session relative to a drug-free, presession baseline phase. The majority of these session changes in firing were decreases. Incrementing the nicotine dose did not significantly affect either the prevalence or average magnitude of the operant- and cue-locked increases in firing. It also had no significant effect on the average firing of the operant- and cue-activated neurons during the SA session. On the other hand, nicotine significantly and dose-dependently increased the prevalence and magnitude of decreases in the average firing of neurons that were nonresponsive during the operant and the cue. The differential effect of nicotine was associated with a net increase in average firing of neurons activated during the nicotine-taking behavior and the nicotine-paired environmental cue relative to the average firing of neurons that were nonresponsive during those events.

### NAc neuronal responses associated with nicotine-taking behavior and nicotine-paired environmental cues

Tobacco smoking in humans is maintained, in part, by sensorimotor cues that occur in conjunction with drug-taking behavior and environmental cues paired with nicotine exposure. The FR1 nicotine SA paradigm closely parallels smoking in humans [2], [52], [59] and establishes conditions under which cues contribute to maintenance of nicotine SA [2], [3], [4], [51]. During the FR1 SA recording session of the present investigation, 14% of neurons showed an increase in firing during either an environmental nicotine-paired cue or both the nicotine-paired cue and the drug-taking behavior. An additional 6% showed an increase in firing exclusively during the drug-taking behavior. Only 2% of recorded neurons showed a decrease in firing during either the cue or the nicotine-taking behavior. A cue-probe session confirmed the specificity of the majority of cue activations. The event-related changes in firing during nicotine SA are consistent with evidence for a role of the NAc in mediating the maintenance of nicotine SA by nicotine-predictive cues. Moreover, the findings support the hypothesis that excitatory neurophysiological mechanisms mediate the NAc role in cue-maintenance of nicotine SA.

### Decrease in average firing of NAc neurons: The acute pharmacological effect of self-administered nicotine on NAc neurons

In the present investigation, NAc neurons exhibited sustained decreases and increases in average firing during both nicotine and sucrose SA. The presence of the session changes in firing during sucrose SA as well as nicotine SA is evidence of a contribution of normal afferent input to the firing patterns. However, a number of observations of the present study are indicative of an additional pharmacological contribution to the decreases in firing during nicotine SA. First, increments in nicotine dose increased the prevalence and magnitude of session decreases during nicotine SA without significantly affecting session increases. Consistent with this observation, incrementing the nicotine dose also decreased the average firing of all NAc neurons combined. Second, control analyses showed that the dose-dependent decrease in average firing was not attributable to nicotine-induced changes in behavior. Third, session decreases were three times more prevalent during nicotine (≥30 µg/inf) sessions compared to sucrose sessions. Moreover, two lines of evidence indicate that the prevalence difference did not reflect a between-group difference in reward magnitude: (1) 32% sucrose is more reinforcing than the training dose of nicotine [46]; yet, in the present study, firing was more depressed during nicotine SA than during sucrose SA; and (2) sucrose concentration (reward magnitude) does not affect the prevalence of session decreases in average firing during FR1 sucrose SA [47]. Together, the findings demonstrate that the pharmacological effect of nicotine on average firing is inhibitory.

The data also show that the inhibitory pharmacological effect of nicotine on NAc neurons is activity-dependent. Specifically, self-administered nicotine significantly and dose-dependently decreased the average firing of neurons that were nonresponsive during the operant and light-tone cue (task-nonactivated neuron group) without significantly affecting the average firing of operant- and cue-activated neurons (task-activated group). Moreover, the differential decrease in average firing did not occur during sucrose SA. In total the observations support an activity-dependent inhibitory effect of self-administered nicotine on average NAc firing.

There have been few electrophysiological investigations of nicotine effects on the NAc. One slice-recording study observed that directly applied nicotine decreased excitatory postsynaptic potentials (EPSPs) of NAc medium spiny neurons, which is consistent with a potential for nicotine-induced inhibition of NAc neuron firing [60]. The effects of drugs *in vitro* do not necessarily predict the effects of drugs in behaving animals. The present findings provide necessary corroborative evidence that the acute effect of self-administered nicotine is indeed inhibitory. Interestingly, the inhibitory effect of nicotine on EPSPs in the slice recording experiment was selective for spontaneous activity and did not impact glutamate-evoked potentials of medium spiny neurons. This activity-dependent inhibitory effect of nicotine on EPSPs is possibly related to the activity-dependent decrease in neuron firing observed in the present study.

The present investigation identified an acute pharmacological effect of self-administered nicotine. However, the findings of the study are also relevant to understanding chronic nicotine effects. The activity-dependent acute effect of nicotine causes a difference in firing rate between the neurons that are activated during nicotine-taking behavior and nicotine-pared cues and neurons that are not activated during those events. The difference in firing rate during drug exposure could make the two neuron groups differentially susceptible to activity-dependent nicotine-induced neuroadaptations. Recent findings support this hypothesis ([46], also see [61], [62]).

### Nicotine-induced amplification of cue effects on nicotine SA

Unconditioned pharmacological effects of nicotine acutely strengthen cue-maintenance of nicotine SA. It has been hypothesized that this nicotine effect is mediated by an increase in the NAc response to nicotine-paired cues [26]. This amplification could involve an absolute increase in either the prevalence or the magnitude of cue-evoked NAc neuronal responses (absolute amplification hypothesis). It could additionally or alternatively involve a relative amplification of the cue responses mediated by a suppression of potentially competing NAc neuronal signals (relative amplification hypothesis). Patterns of NAc neuronal activity reminiscent of the hypothesized mechanisms can be found in previous studies of drug and natural reward [63], [64], [65], [66].

In this investigation, nicotine dose had no significant net effect on the prevalence and average magnitude of neuronal responses during either nicotine-taking behavior or the nicotine-paired environmental cue. The present findings thus do not support the hypothesis that nicotine has an overall amplifying effect on NAc neural responses during those events. However, individual neuron analyses showed that an increase in nicotine dose was associated with an increment in the response magnitude of a subset of neurons activated during the cue (average increase >65%). On the basis of this observation, it would be worthwhile to test for a selective absolute amplification of neural responses in additional experiments designed to differentiate functionally distinct subtypes of cue-responsive neurons.

The data of this investigation were straightforward with respect to the relative amplification hypothesis. Nicotine did not significantly affect average firing of neurons activated during the drug-taking behavior and the environmental nicotine-paired cue, but it significantly decreased the average firing rate of neurons nonresponsive during those events. The activity-dependent nicotine effect was associated with a significant net increase in the firing of the activated neurons relative to that of the nonresponsive neurons. This observation supports the relative amplification hypothesis.

The similar response of cue- and operant-activated neurons to nicotine points to a potential role for both types of neurons in cue-maintained nicotine SA and nicotine-induced amplification of those cue effects. This hypothesis remains to be tested; however, it is feasible, given that both environmental cues and sensorimotor cues associated with drug-taking play a role in maintaining nicotine SA.

### Comparisons to cocaine SA recording studies

Previous recording studies have characterized the activity of NAc neurons during FR1 cocaine SA. Comparison of the findings of those studies to the data of the present investigation show that the firing patterns exhibited by NAc neurons during nicotine and cocaine SA are similar in a number of respects: A subset of neurons shows phasic increases in firing time-locked to the drug-taking behavior and drug-paired environmental cues [48], [49], [67], [68]; operant- and cue-locked NAc neuronal responses are stronger in NAc core compared to NAc shell [67], [69]; and more than 50% of neurons exhibit a change in average firing rate during the SA session compared to a drug-free, presession baseline period, the majority of which are decreases [56], [70], [71]. The decreases but not the increases in firing are pharmacological. In addition the decreases are activity-dependent, impacting phasically activated neurons significantly less than neurons that are nonactivated during the SA session. The differential decrease in firing is associated with a net increase in average firing of neurons activated during drug-taking behavior and drug-paired environmental cues relative to the average firing of neurons that are nonresponsive during those events [47], [72]. Overall, the nicotine and cocaine data suggest substantial overlap between the neurophysiological events that mediate the NAc role in nicotine- and cocaine-directed behavior (though see Supplementary Information **S1** **: Nicotine SA: Long-duration phasic changes in firing during the ITI**).

The NAc firing patterns observed during sucrose SA in this study are comparable to those observed in other recording studies of nondrug rewards conducted in rats [46], [57], [69], [73]. The firing patterns are also similar in a number of respects to those observed during nicotine and cocaine SA. Nevertheless, comparisons of sucrose and drug (nicotine or cocaine) SA show two reliable differences in NAc firing patterns.

First, more phasic decreases are time-locked to the reinforced lever-press operant during sucrose SA compared to either nicotine or cocaine SA. One factor that might explain this difference in phasic responses is a between-reward difference in reward approach and reward consumption, which are associated predominantly with inhibitory NAc neural responses and occur during food but not drug SA [74], [75], [76], [77], [78], [79]. Consistent with this explanation, the prevalence of phasic decreases in firing time-locked to the reinforced operant is similar between oral ethanol SA and sucrose SA [80], [81].

A second reliable difference in NAc firing patterns between sucrose and drug (nicotine or cocaine) SA is a lower prevalence of session decreases during sucrose SA (including SA of maximally reinforcing sucrose concentration) compared to drug SA. Available evidence indicates that this between-reward difference reflects the pharmacological actions that occur uniquely during drug SA (present investigation, [47] and might thus be of particular importance to differences in behavior directed toward drug and food rewards.

### Conclusions

The present findings support two hypotheses about the neurophysiological mechanisms that mediate nicotine SA. First, phasic increases in firing mediate the role of the NAc in cue-maintenance of nicotine SA. Second, nicotine-induced amplification of cue-maintenance of nicotine SA involves an activity-dependent, nicotine-induced decrease in average NAc firing and a relative amplification of NAc neuronal signals during nicotine-taking behavior and nicotine-paired environmental cues. In addition, the present investigation showed that the NAc firing patterns during nicotine SA are similar to those that occur during cocaine SA. This observation is suggestive of overlap between the NAc neurophysiological mechanisms that mediate nicotine- and cocaine-directed behavior. Finally, observation of an activity-dependent acute pharmacological effect of self-administered nicotine has implications for understanding chronic effects of nicotine SA.

## Supporting Information

Figure S1 **Long-duration phasic change in firing time-locked to the reinforced operant during the FR1 nicotine SA session.** In the histogram, average firing rate (Hz per 0.1-min bin) of a single neuron is plotted during the 4 min before and after the reinforced press (last 15 presses). Time 0 = completion of the cocaine-reinforced lever-press.
(TIF)

Figure S2 **Distribution of core versu** ***s*** **shell neurons.** (**A**) Number of core and shell neurons recorded during nicotine and sucrose SA. (**B**) Number of task-activated and task-nonactivated neurons recorded in the core and shell during nicotine and sucrose SA. (**C**) Number of core and shell neurons recorded during nicotine FR1 SA, cue-probe, and nicotine dose-response sessions.
(TIFF)

Figure S3 **Phasic increases that occurred during the operant and the cue period: effect of nicotine dose.** (**A–B**) Average prevalence (A) and magnitude (B) of phasic increases in firing during the operant (black bars) and the light-tone cue (white bars) plotted as a function of nicotine dose.
(TIF)

Figure S4 **Average firing of task-activated and task-nonactivated neurons during different behavioral periods.** (**A**) Average firing of task-activated and task-nonactivated neurons during the baseline phase (Baseline) and three behavioral periods during the FR1 SA session. The behavioral periods are −12 to −9 s prepress period (Background), the 1-s operant period (operant), and the 1-s cue period (cue). (**B**) Average firing of task-activated and task-nonactivated neurons during three behavioral periods during the nicotine dose-response session. The behavioral periods are the same as those shown for the FR1 SA session in panel A: −12 to −9 s prepress (Background), and the 1-s operant and cue periods (operant and cue, respectively).
(TIFF)

Figure S5 **Core versus shell: Prevalence and magnitude of phasic increases in firing time-locked to the nicotine-reinforced operant during the FR1 SA session.** (**A**) Average prevalence of phasic responses is shown for the shell and the core. (**B**) Average magnitude of phasic responses is shown for the shell and the core. *p<0.05, significant difference between groups.
(TIFF)

Figure S6 **Core versus shell: Changes in average firing during the nicotine FR1 SA session.** (**A**) Prevalence of session-decrease and session-increase firing patterns during the nicotine FR1 SA session is shown for shell and core. *p<0.05, significant difference in overall prevalence of session-decrease and increase firing patterns (no significant effect of subterritory on prevalence of firing patterns). (**B**) Average firing rate during the presession baseline and SA phases is shown for shell and core. (**C**) Average firing rate of task-activated and task-nonactivated neurons during the presession baseline (Baseline) and SA (Session) phases is shown for shell (left panel of C) and core (right panel of C).
(TIFF)

Supplementary Information S1 **Description of supporting control analyses.**
(DOCX)
