# Supplementary figures and images for: Acute Effects of Nicotine Amplify Accumbal Neural Responses during Nicotine-Taking Behavior and Nicotine-Paired Environmental Cues
Source: PLoS One. 2011 Sep 22;6(9):e24049. doi: 10.1371/journal.pone.0024049 (PMC3178519; doi:10.1371/journal.pone.0024049)

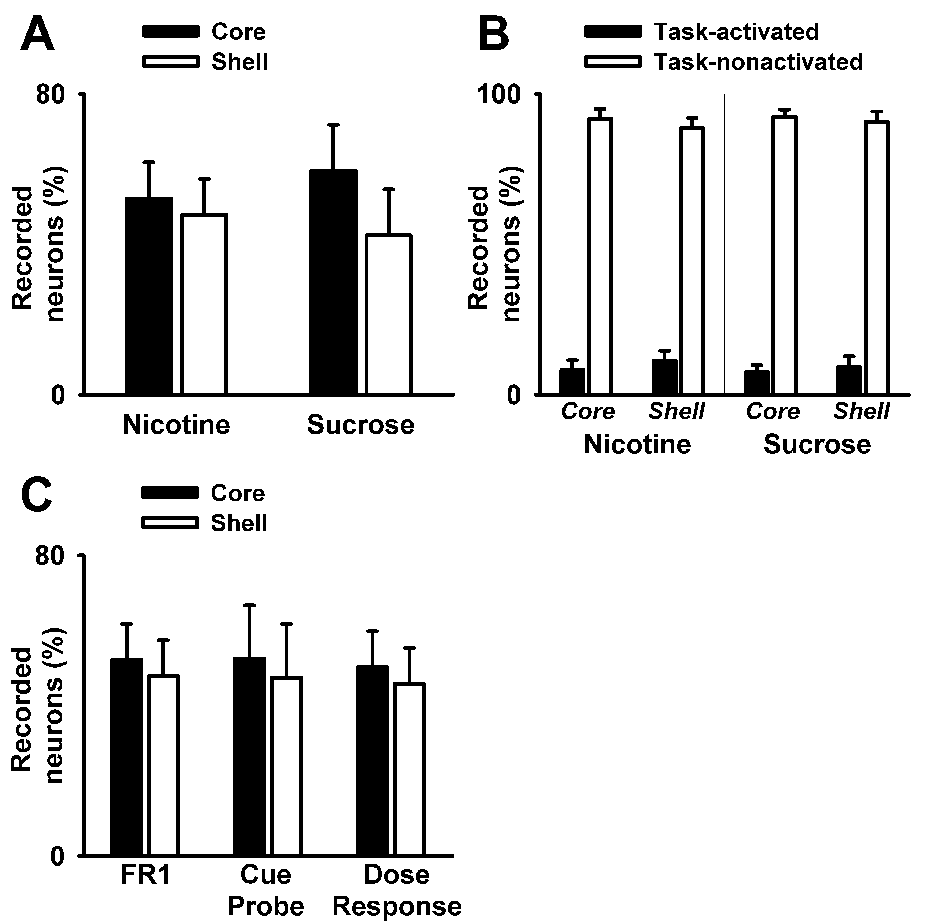

Supplement: Figure S2 — Distribution of core versu s shell neurons. (A) Number of core and shell neurons recorded during nicotine and sucrose SA. (B) Number of task-activated and task-nonactivated neurons recorded in the core and shell during nicotine and sucrose SA. (C) Number of core and shell neurons recorded during nicotine FR1 SA, cue-probe, and nicotine dose-response sessions. (TIFF) [file pone.0024049.s002.tiff]

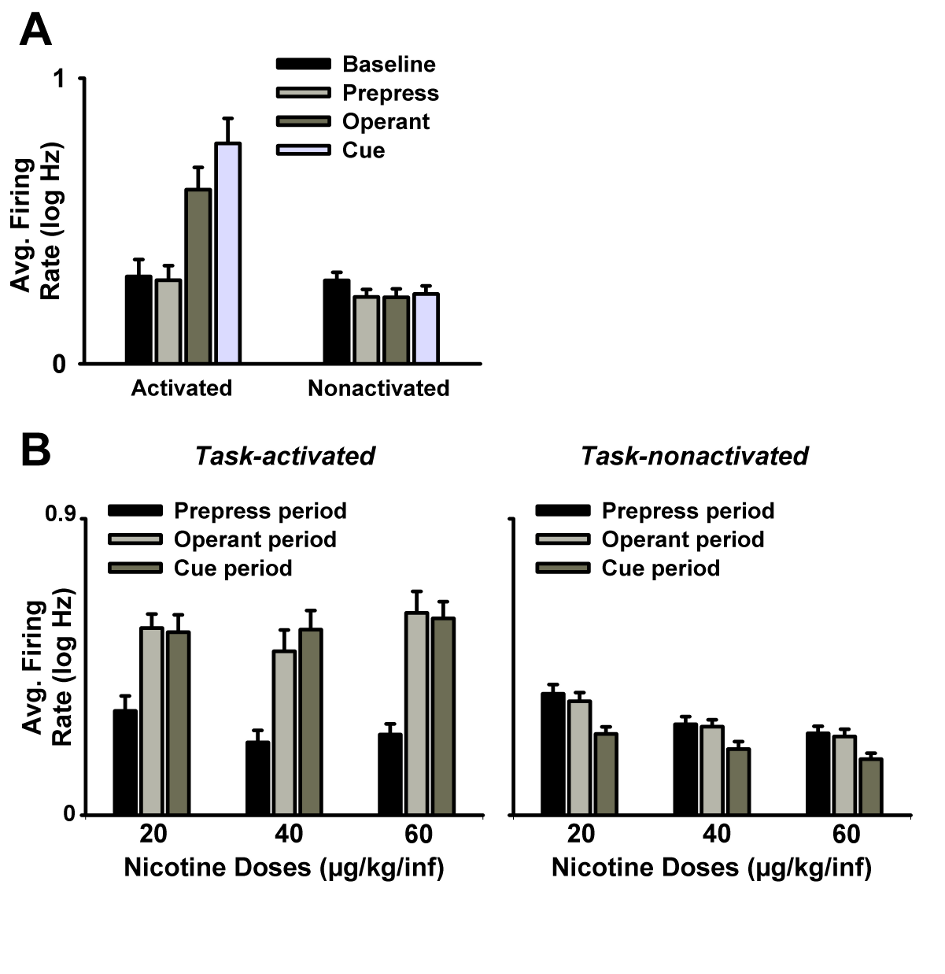

Supplement: Figure S4 — Average firing of task-activated and task-nonactivated neurons during different behavioral periods. (A) Average firing of task-activated and task-nonactivated neurons during the baseline phase (Baseline) and three behavioral periods during the FR1 SA session. The behavioral periods are −12 to −9 s prepress period (Background), the 1-s operant period (operant), and the 1-s cue period (cue). (B) Average firing of task-activated and task-nonactivated neurons during three behavioral periods during the nicotine dose-response session. The behavioral periods are the same as those shown for the FR1 SA session in panel A: −12 to −9 s prepress (Background), and the 1-s operant and cue periods (operant and cue, respectively). (TIFF) [file pone.0024049.s004.tiff]

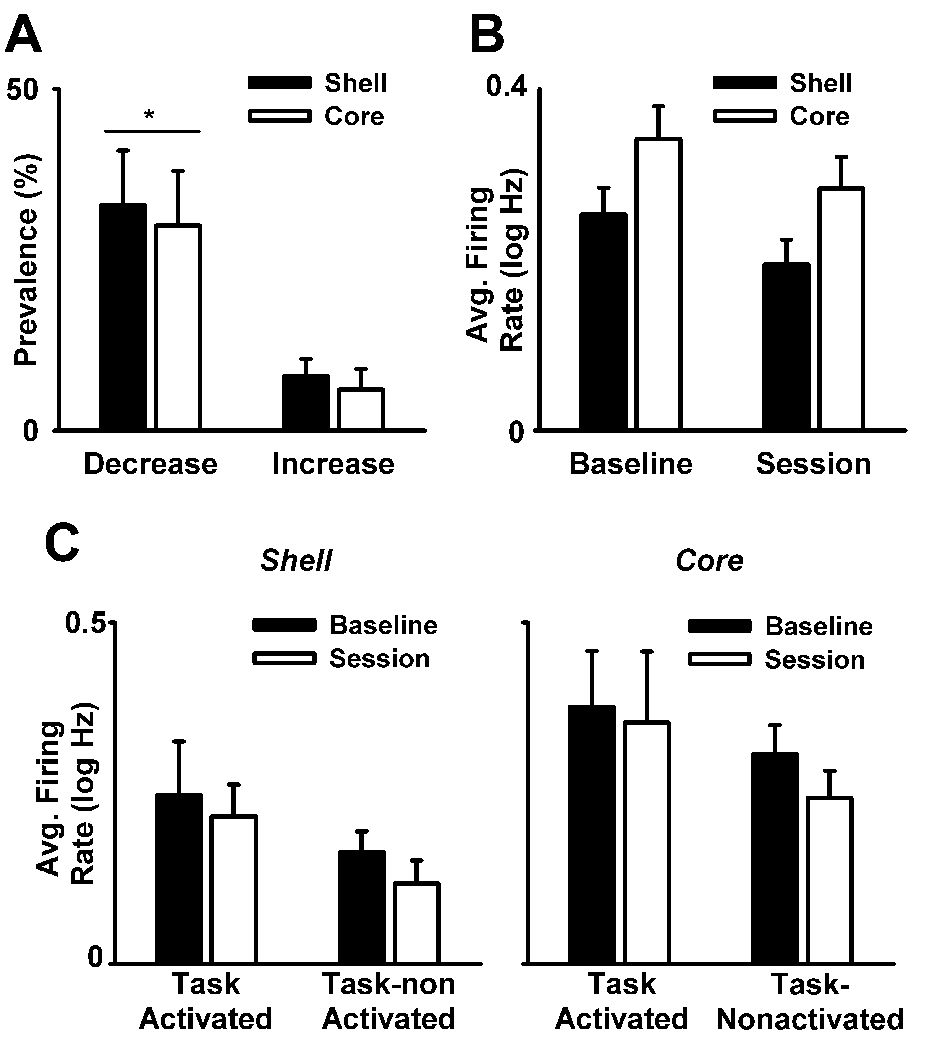

Supplement: Figure S6 — Core versus shell: Changes in average firing during the nicotine FR1 SA session. (A) Prevalence of session-decrease and session-increase firing patterns during the nicotine FR1 SA session is shown for shell and core. *p<0.05, significant difference in overall prevalence of session-decrease and increase firing patterns (no significant effect of subterritory on prevalence of firing patterns). (B) Average firing rate during the presession baseline and SA phases is shown for shell and core. (C) Average firing rate of task-activated and task-nonactivated neurons during the presession baseline (Baseline) and SA (Session) phases is shown for shell (left panel of C) and core (right panel of C). (TIFF) [file pone.0024049.s006.tiff]
