# Supplementary material for: Acute Effects of Nicotine Amplify Accumbal Neural Responses during Nicotine-Taking Behavior and Nicotine-Paired Environmental Cues
Source: PLoS One. 2011 Sep 22;6(9):e24049. doi: 10.1371/journal.pone.0024049 (PMC3178519; doi:10.1371/journal.pone.0024049)
Supplement: Supplementary Information S1 — Description of supporting control analyses. (DOCX) [file pone.0024049.s007.docx]

**Supplementary Materials**

**Nicotine self-administration: long-duration phasic changes in firing during the intertrial interval**

In a number of previous studies of cocaine self-administration (SA) [1,2,3,4], researchers documented that nucleus accumbens (NAc) neurons exhibit long-duration changes in average firing time-locked to the cocaine-reinforced operant. Some of these changes in firing occur during the ~0.5-min pre- and post-press (Figure 3 in [3]). Other long-duration changes in firing (about half of all recorded neurons) occur over the course of the entire intertrial interval. Most of those latter changes in firing involve a decrease in firing during the 1-min postpress followed by a progressive return to the prepress firing rate shortly before the next operant response (referred to as decrease + progressive reversal firing pattern) (Figure 2 in [3]).

We tested for long-duration phasic changes in firing during the FR1 SA session of the present study. The firing rate for each neuron was calculated during the 2-min pre- and postpress using a 0.5-min sliding window (with a 0.1-min step). A change in firing was defined as a significant difference in firing between maximum and minimum firing rates (during a 0.5 min period) during the 2.0 min pre- versus postpress (see [3] for details). Similar tests were conducted for neurons recorded during the dose-response session. During the fixed ratio 1 (FR1) nicotine SA session, only 2 neurons showed a long-duration change in firing and neither of these changes was of the decrease + progressive reversal type (Figure S1). During the dose-response session, a significant 0.5-min pre- and postpress change in firing was exhibited by 1 neuron at the 40 µg/kg/inf dose and by 2 neurons at the 60 µg/kg/inf dose. The changes in firing that occurred during the dose-response session were similar to the change shown in Figure S1.

During cocaine SA sessions, the timing of cocaine self-infusions is determined by drug level [5,6,7,8]. The decrease + progressive reversal firing pattern exhibited by NAc neurons during cocaine SA is inversely correlated with drug level and positively correlated with the timing of cocaine-reinforced operant responses. Given these and other findings, the firing pattern has been hypothesized to contribute to NAc mediation of ongoing cocaine SA [2,3,4]. Though the reason for the differential presence of the long-duration firing patterns during nicotine and cocaine SA is not known, it is interesting to note that in both animals and humans, nicotine SA generally appears less regulated by drug level compared to SA of other abused drugs, including cocaine [9,10,11,12].

**Comparable between-reward and between-session distribution of neurons in the NAc core and shell**

#### Tests were conducted to confirm that the relative prevalence of core and shell neurons was similar between the nicotine and sucrose groups during the FR1 SA session. A between-group ANOVA performed on the percentage of recorded neurons with reward (nicotine *vs* sucrose) and subterritory (core *vs* shell) as factors showed no significant effect of reward (*F*_(1,32)_ = 0.33; NS) and no significant interaction between reward and subterritory (*F*_(1,32)_ = 1.19; NS) (Figure S2A). A similar analysis was conducted to test for a differential distribution of task-activated versus task-nonactivated neurons between the core and the shell. A between-group ANOVA with reward, subterritory, and activation (task-activated *vs* task-nonactivated) as factors also showed no significant effect of reward (*F*_(1,32)_ = 0.05; NS), no significant interaction between reward and subterritory (*F*_(1,32)_ = 1.84; NS), and no significant interaction among reward, subterritory, and activation (*F*_(1,32)_ = 0.27; NS) (Figure S2B). These findings show that the differences in NAc firing patterns between the nicotine and sucrose groups cannot be attributed to a difference in the percentage of core versus shell neurons.

#### Tests were also conducted to determine whether the sampling of core and shell neurons was stable across successive recording sessions in the nicotine group. A between-group ANOVA performed on the percentage of recorded neurons with recording session (FR1 SA, cue-probe, and dose-response sessions) and subterritory as factors showed no significant effect of recording session (*F*_(2,54)_ = 1.24; NS) and no significant interaction between recording session and subterritory (*F*_(2,54)_ = 0.64; NS) (Figure S2C). This analysis shows that the percent of core versus shell neurons recorded in the nicotine group was stable across repeated recording sessions.

**Nicotine dose-response session**

***Comparable effects of nicotine dose on the prevalence and magnitude of phasic increases in firing during the nicotine-taking behavior versus the light-tone cue*.** Phasic increases in firing during the operant and the light-tone cue potentially reflect neuronal activity that is differentially susceptible to nicotine effects. It was not possible to test for an effect of operant versus cue period in a single ANOVA analysis of phasic activity, as some individual neurons exhibited phasic responses during both periods and thus could not be assigned to a single treatment level of the period factor in the primary ANOVA analysis. Therefore, to test for a possible selective effect of nicotine on operant versus cue responses, we repeated the original ANOVA analyses of prevalence and response magnitude, separately for neurons showing a response during the operant period and for neurons showing a response during the cue period. A repeated-measures ANOVA applied to all operant-responsive neurons (the operant neurons and the operant + cue neurons) with dose as a factor (20, 40, and 60 µg/kg/inf) showed no significant effect of dose on either the prevalence (*F*_(2,16)_ = 0.04; NS) (Figure S3A) or the magnitude (*F*_(2,10)_ = 1.08; NS) (Figure S3B) of increases in firing rate during the operant. Similarly, a repeated-measures ANOVA applied to all cue-responsive neurons (the cue neurons and the operant + cue neurons) showed no significant effect of dose on either the prevalence (*F*_(2,16)_ = 0.21; NS) (Figure S3A) or the magnitude (*F*_(2,14)_ = 0.56; NS) (Figure S3B) of increases in firing during the cue. Thus, increases in nicotine dose did not significantly affect phasic increases in firing during either the operant or the cue period.

#### The differential decrease in average firing rate between task-activated and task-nonactivated neurons during the FR 1 SA session is not specific to a particular behavior

A control analysis was conducted to test the behavioral generality of the activity-dependent effect on average firing rate during the SA session. The average firing rate during the SA session was determined for task-activated and task-nonactivated neurons during the presession baseline period and for each of three behavioral periods (−12 to −9 s before the reinforced press, the operant period, and the cue period). A repeated-measures ANOVA applied to those average firing rates, with activation and behavioral period (presession baseline, −12 to −9 s prepress, operant period, and cue period) as factors, showed a significant effect of activation (*F*_(1,76)_ = 17.74; *p* < 0.001), a significant effect of behavioral period (*F*_(3,228)_ = 37.18; *p* < 0.001), and a significant interaction between activation and behavioral period (*F*_(3,228)_ = 43.40; *p* < 0.001). Post hoc analysis showed that the average firing rate of task-nonactivated neurons during the −12 to −9 s prepress period, the operant period, and the cue period was significantly decreased compared to the average firing rate during the presession baseline phase (*p* < 0.01, *p* < 0.01, and *p* < 0.05, respectively). The average firing rate of the task-activated neurons was not significantly decreased between baseline and any of the three behavior periods (Figure S4A)**.** These findings show that the differential decrease in average firing exhibited by the task-activated and task-nonactivated group was a behaviorally general effect.

#### The differential decrease in average firing between task-activated and task-nonactivated neurons during the dose-response session is not specific to a particular behavior

Control analyses tested the behavioral generality of the effect of nicotine dose on the average firing of the task-activated and task-nonactivated neurons. The average firing rate of each group was calculated during each of three behavioral periods (−12 to −9 s before the reinforced press, the operant period, and the cue period). An ANOVA applied to these firing rates with group, dose, and behavioral period as factors showed a significant effect of dose (*F*_(2,114)_ = 5.96; *p* < 0.01) and a significant interaction between dose and activation (*F*_(2,114)_ = 2.94; *p* < 0.05), but no significant interaction either between behavioral period and dose (*F*_(4,228)_ = 0.29; NS) or among behavioral period, dose, and activation (*F*_(4,228)_ = 0.82; NS). Post hoc analysis showed that the average firing rate of the task-nonactivated group during the three behaviors decreased dose-dependently (20 *vs* 40, *p* < 0.05; 20 *vs* 60, *p* < 0.01), whereas the average firing rate of the task-activated neuron group showed no significant change (Figure S4B). This analysis shows that the differential susceptibility of task-activated and task-nonactivated neurons to inhibitory effects of nicotine was not specific to a particular behavior and was not a secondary effect of a nicotine-induced change in behavior.

**Nicotine FR1 SA session: core versus shell comparisons**

***Phasic firing patterns*.** There was a trend for phasic increases time-locked to the nicotine-reinforced press to be greater in prevalence and magnitude in the core relative to the shell during the FR1 SA session. Between-group ANOVAs showed that the difference in magnitude was significant (*F*_(1,18)_ = 4.63; *p* < 0.05) (Figure S5) but that the difference in prevalence was not (*F*_(1,18)_ = 0.76; NS) (Figure S5).

***Session-change firing patterns*.** To test for an effect of subterritory on session changes in firing, we compared the prevalence of the session changes as well as the average firing rates of neurons recorded in the 2 subterritories. A mixed-design ANOVA applied to the prevalence of the firing patterns (average number per animal), with subterritory and firing pattern (session-increase *vs* session decrease) as factors, showed a significant effect of firing pattern but no significant effect of subterritory (*F*_(1,36)_ = 0.19; NS) on the prevalence of session-increase and session-decrease firing patterns and no significant interaction between subterritory and firing pattern (*F*_(1,36)_ = 0.02; NS) (Figure S6A). An analysis of the average firing rates during the presession baseline and SA phase, with session phase (presession baseline *vs* SA phase) and subterritory as factors, showed a significant effect of subterritory (*F*_(1,85)_ = 3.66; *p* < 0.01) and a significant effect of session phase (*F*_(1,85)_ = 11.37; *p* < 0.01) but no significant interaction between subterritory and session phase (*F*_(1,85)_ = 0.30; NS). The ANOVA results show that the average firing rate of shell neurons was lower than the average firing rate of core neurons. However, the average firing rate underwent a similar decrease during SA relative to the presession baseline in the 2 subterritories (Figure S6B).

An additional analysis was conducted to test for an effect of subterritory on the differential decrease in average firing of task-activated versus task-nonactivated neurons. An ANOVA applied to average firing with activation (task-activated *vs* task-nonactivated), session phase (presession baseline *vs* session), and subterritory as factors showed a significant effect of activation (*F*_(1,74)_ = 6.32; *p* < 0.05) and a significant effect of subterritory (*F*_(1,74)_ = 4.30; *p* < 0.05). The effect of subterritory reflected overall lower firing rates in the shell than in the core (Figure S6B and S6C *vs* S6D). The analysis also showed no significant interaction between subterritory and activation (*F*_(1,74)_ = 0.69; NS); no significant interaction between subterritory and session phase (*F*_(1,74)_ = 0.66; NS); and no significant interaction among subterritory, activation, and session phase (*F*_(1,74)_ = 0.52; NS) (Figure S6C). Thus, the activity-dependent decrease in average firing of NAc neurons occurred similarly in the core and the shell.

**Sucrose FR1 SA session**

The characterization of average firing-rate changes during sucrose SA was repeated with the phasic-decrease neurons excluded. As in the original analysis of the sucrose FR1 SA session, the prevalence of session decreases and increases in firing did not differ significantly (15/50 *vs* 20/50 respectively, *F*_(1,14)_ = 2.61; NS). The same was true for the magnitude of the session decreases and increases (0.41 ± 0.06 *vs* 0.38 ± 0.06, respectively; *F*_(1,18)_ = 0.15; NS). Consistent with these observations, the average firing rate of all neurons recorded during the sucrose FR1 SA session, excluding the phasic-decrease neurons, did not undergo a significant change during the SA phase relative to the presession baseline phase (0.42 ± 0.04 during baseline phase *vs* 0.41 ± 0.04 log Hz during sucrose SA phase; *F*_(1,49)_ = 0.03; NS). These analyses showed that, during the FR1 SA session, the between-reward (nicotine *vs* sucrose) difference in prevalence of session decreases was not attributable to a difference in phasic-decrease firing patterns. The finding supports a pharmacological interpretation of the between-reward difference in session decreases in firing.

**References**

1. Peoples LL, Bibi R, West MO (1994) Effects of intravenous selfadministered cocaine on single cell activity in the nucleus accumbens of the rat. Problems of drug dependence, 1993: proceedings of the 55th annual scientific meeting The College on Problems of Drug Dependence, Inc Vol II,. Washington, D.C.: Superintendent of Documents, United States Government Printing Office. National Institute on Drug Abuse Research Monograph 141.: Harris L, ed. pp. pp 326.

2. Peoples LL, Gee F, Bibi R, West MO (1998) Phasic firing time locked to cocaine self-infusion and locomotion: dissociable firing patterns of single nucleus accumbens neurons in the rat. J Neurosci 18: 7588-7598.

3. Peoples LL, West MO (1996) Phasic firing of single neurons in the rat nucleus accumbens correlated with the timing of intravenous cocaine self-administration. J Neurosci 16: 3459-3473.

4. Nicola SM, Deadwyler SA (2000) Firing rate of nucleus accumbens neurons is dopamine-dependent and reflects the timing of cocaine-seeking behavior in rats on a progressive ratio schedule of reinforcement. J Neurosci 20: 5526-5537.

5. Seevers MH, Schuster CR (1967) Self-administration of psychoactive drugs by the monkey: a measure of psychological dependence. Science 158: 535.

6. Pickens R, Thompson T (1968) Cocaine-reinforced behavior in rats: effects of reinforcement magnitude and fixed-ratio size. J Pharmacol Exp Ther 161: 122-129.

7. Yokel RA, Piekens R (1974) Drug level of d- and l-amphetamine during intravenous self-administration. Psychopharmacologia 34: 255-264.

8. Gerber GJ, Wise RA (1989) Pharmacological regulation of intravenous cocaine and heroin self-administration in rats: a variable dose paradigm. Pharmacol Biochem Behav 32: 527-531.

9. Goldberg SR, Spealman RD, Risner ME, Henningfield JE (1983) Control of behavior by intravenous nicotine injections in laboratory animals. Pharmacol Biochem Behav 19: 1011-1020.

10. Risner ME, Goldberg SR (1983) A comparison of nicotine and cocaine self-administration in the dog: fixed-ratio and progressive-ratio schedules of intravenous drug infusion. J Pharmacol Exp Ther 224: 319-326.

11. Rose JE, Corrigall WA (1997) Nicotine self-administration in animals and humans: similarities and differences. Psychopharmacology (Berl) 130: 28-40.

12. Lynch WJ, Carroll ME (1999) Regulation of intravenously self-administered nicotine in rats. Exp Clin Psychopharmacol 7: 198-207.
